# Quantitative evaluation of apically extruded debris using TRUShape, TruNatomy, and WaveOne Gold in curved canals

**DOI:** 10.1038/s41405-022-00106-8

**Published:** 2022-05-20

**Authors:** Nehal Nabil Roshdy, Reham Hassan

**Affiliations:** 1 Department of Endodontics, Faculty of Dentistry, Cairo University, Giza, Egypt; 2 Department of Endodontics, Faculty of Dentistry, Minia Univeristy, Giza, Egypt; 3 Department of Endodontics, Faculty of Dentistry, Egyptian Russian University, Badr city, Egypt

**Keywords:** Endodontic files, Dentistry

## Abstract

**Objective:**

This study compared the quantity of extruded debris after instrumentation with TRUShape 3D Conforming files, TruNatomy files, and the WaveOne Gold reciprocating system.

**Materials and methods:**

Fifty-one mesiobuccal canals with severe curvatures (25–40°) were assigned to three equal groups according to the rotary system used for preparation, either TRUShape, TruNatomy, or WaveOne Gold files. The extruded debris was collected in pre-weighed glass vials. The data were statistically analyzed using a one-way ANOVA test and Tukey’s post hoc test.

**Results:**

The least extruded debris was obtained with the WaveOne Gold instruments compared to the TRUShape and TruNatomy files (*p* < 0.001).

**Conclusions:**

Debris extrusion occurs independently of the motion or design of the instrument. The WaveOne Gold system outperformed TRUShape and TruNatomy files in this study.

## Introduction

Root canal treatment aims to thoroughly debride the root canal space, allowing the canal to be shaped and filled with an inert obturating material, thus blocking or reducing any venues of reinfection [1]. Despite attempts to maintain the proper working length during the mechanical preparation phase, debris in the form of pulp fragments, microorganisms, dentinal chips, necrotic remnants, and irritants are inevitably pushed out from the apex toward the periapical tissues [2]. This occurrence is significant because the extruded material may elicit an inflammatory reaction in the apical tissues causing postoperative pain or flare-ups [3, 4].

Variations in mechanical preparation techniques influence the amount of debris extruded. Balanced force and crown-down techniques are associated with reduced quantities of extruded debris compared to a linear filing motion [5]. Rotary instruments result in less extruded debris when compared to hand files [6]. Rotary instruments tend to draw the debris toward their flutes, guiding debris in a coronal direction out of the canal space [7].

There has been a recent trend of shifting toward preserving tooth structure in terms of reducing the amount of dentin removed during root canal treatment. This treatment protocol means cutting smaller access cavities, preserving the pericervical dentin, avoiding aggressive dentine removal, and maintaining the natural canal anatomy during shaping [8]. Nickel–titanium (NiTi) rotary instruments with improved metallurgy aiming to achieve minimally invasive endodontics with maximum preservation of radicular dentin are currently available in the market.

TRUShape 3D conforming file (TRS; Dentsply Sirona, Tulsa, OK, USA) has an inventive design with an S-shape curve in the longitudinal axis, which provides an increased contact with the canal surfaces. According to the manufacturer, the instrument’s symmetrical triangular cross-section allows for better dentin conservation during canal shaping, adhering to the principle of minimally invasive endodontics and conserving the root structure’s integrity [9]. The TRS system includes different sizes and tapers: 20/0.06 v, 25/0.06 v, 30/0.06 v, and 40/0.06 v, where 0.06 v refers to the taper in the apical 2 mm and the variable taper along the working part of the instrument.

TruNatomy (TRN; Dentsply Sirona, Maillefer, Ballaigues, Switzerland) files have been manufactured from thin 0.8 mm NiTi wire rather than the traditional 1.2 mm NiTi wire used to fabricate most standard files. They are then exposed to a special heat treatment. The TRN system consists of an orifice modifier, a glider with a centered cross-section parallelogram design, and shaping files available in three sizes, small (20/0.04 taper), prime (26/0.04 taper), and medium (36/0.03 taper) with an off-centered parallelogram cross-sectional design [10]. The manufacturer claims that TRN files offer slim shaping and enhanced debridement due to the additional space created by the file’s unique design. The TRN system conserves the tooth’s integrity with the maximum preservation of pericervical dentine due to the slim design, instrument geometry, regressive tapers, and the heat treatment of the alloy [10, 11].

WaveOne Gold (WOG; Dentsply Sirona, Ballaigues, Switzerland) files perform root canal preparation using a single instrument with a reciprocating movement. They are manufactured from a new proprietary super metal technology termed “Gold-wire,” producing a super-elastic NiTi file. This system includes four tip sizes: small (20/0.07), primary (25/0.07), medium (35/0.06), and large (45/0.05). Each file has a parallelogram off-centered cross-section with 85° cutting edges in contact with the canal and a variable, reducing taper. It operates at a speed of 350 rpm (150° CCW and 30° CW direction), completing 360° in three cycles [12].

Currently, limited evidence exists on the effect of minimally invasive canal-shaping procedures using TRS and TRN instruments on the quantity of apically extruded debris. Thus, this investigation aimed to compare the amount of apically extruded debris after the preparation of severely curved mesiobuccal canals of extracted mandibular first molars using these three minimally invasive canal-shaping systems. The null hypothesis tested was that there will be no difference in the amount of extruded debris between TRUShape, TruNatomy, and WaveOne Gold files.

## Materials and methods

### Sample size

By adopting an alpha (*α*) level of 0.05 (5%), a beta (*β*) level of 0.20 (20%), i.e., power = 80%, and effect size (*f*) of 0.760, calculated based on the results of Boijink et al. [13]; the predicted sample size was a total of 51 samples. Sample size calculation was performed using G*Power version 3.1.9.7.

### Sample selection

After the ethical committee at the Faculty of Dentistry of Cairo University in Egypt (no.1739) approved the study protocol, freshly extracted mandibular molars were collected from the university’s Department of Oral Surgery. The teeth were cleaned from calculus and debris and examined under a surgical operating microscope (G6, Global Surgical Corp; USA) for caries, fractures, calcifications, cracks, or resorptions in their mesial roots. The teeth were placed in 5.25% NaOCl (Clorox Co, 10th of Ramadan, Egypt) for 10 min to remove soft debris and were then stored in saline until use.

Fifty-one mesial roots from the mandibular molars were chosen with mesiobuccal canals having curvature angles between 25° and 45° measured using the Schneider method [14] and a curvature radius of less than 6 mm, which was assessed using image analysis software (OnDemand 3D software; CBCT: Scanora 3D, Soredex, Finland). Periapical radiographs were taken in mesiodistal and buccolingual directions. Mesial roots with type IV Vertucci canal configuration were selected. Teeth with external defects, calcification, and Vertucci type II were excluded. To ensure homogeneity between the groups, the teeth were grouped according to the canal curvature angle and radius [15].

The buccal cusp tip of each tooth was flattened to act as a reference point. The teeth were accessed using high‐speed diamond burs (1012 HL; KG Sorensen). A diamond saw mounted on a low-speed micromotor was used to separate the distal from the mesial roots. The patency in mesiobuccal canals was verified by inserting a 10 K file (Dentsply Sirona, Tulsa, OK) into the canal space until the tip was visible at the apical foramen. The working length was calculated by subtracting 1 mm from this measurement. Working lengths were adjusted to 17 mm in all the canals to eliminate any confounders that might affect the results.

### Instrumentation and debris collection model

**Fig. 1:**
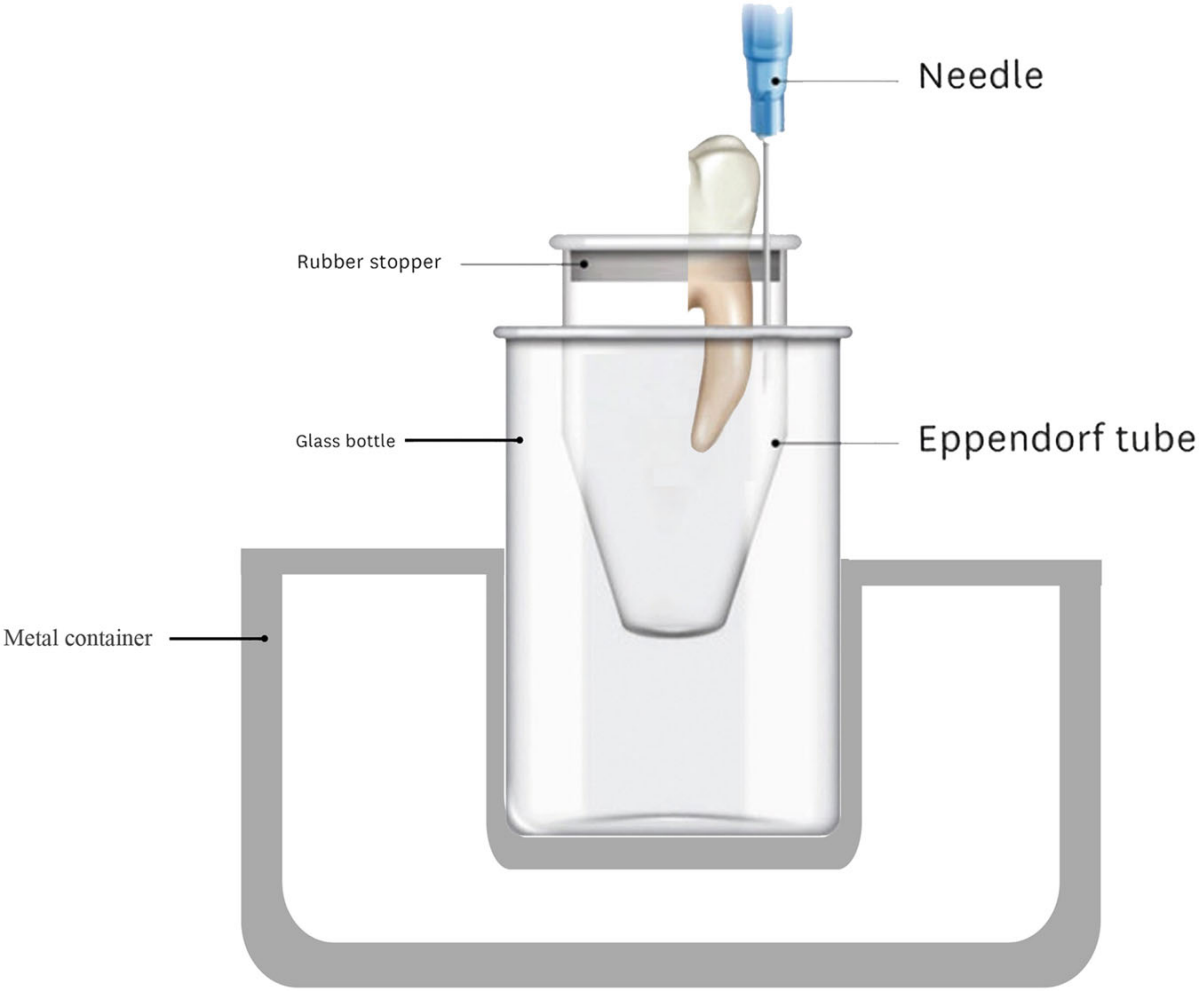
Schematic representation of the debris collection test model. The mesial root was inserted into a hole made on the stopper of an empty eppendorf tube. To keep the air pressure inside and outside of the tubes balanced, a 27-gauge bent needle was inserted. The whole apparatus was concealed in a glass vial covered with adhesive plaster to prevent the operator from seeing through during the instrumentation process. The whole assembly was mounted in a metal container.

The process used for collecting the apically extruded debris was adopted from a technique previously described by Myers and Montgomery [16]. The external root surfaces were covered with a double layer of nail polish except for 1 mm around the apex. Empty Eppendorf tubes were numbered and weighed without the stoppers using an analytical balance (Radwag, Radom, Poland) with a precision of 10^5^ to measure the pre-experimental weights of the tubes. Each tube was measured without stoppers three times, and the mean values of these measurements were calculated and noted as the initial weight (W1). A hole was made on the stoppers of each of the tubes using a hot instrument. The mesial roots were inserted into these holes under pressure, and a 27-gauge bent needle (Genject, Ankara, Turkey) was inserted together with the stopper to keep the air pressure inside and outside of the tubes balanced. The teeth were affixed to the stoppers with cyanoacrylate (Sapheon Inc, Santa Rosa, CA, USA). The stoppers were attached to their Eppendorf tubes, and the whole apparatus was then concealed in a glass vial covered with adhesive plaster to prevent the operator from seeing through during the instrumentation process. The whole assembly was mounted to prevent any movement, ensuring the standardization of the instrumentation procedure and avoiding any direct contact with the vials (Fig. 1). The vials were coded and allocated using a random group allocation online software (https://www.ramdomizer.org) into three groups (*n* = 17 per group) according to the file system used.

Group I: rotary instrumentation with TRUShape 3D conforming files (TRS). The mesiobuccal canal was mechanically prepared with TRS orifice modifier 20/0.08 followed by TRS file 25/0.06 with a red ring using the X-Smart Plus endodontic motor (Dentsply Sirona, Tulsa, OK) at a speed of 300 rpm and a 3 N/cm torque to shape the middle third, with a 2–3 mm amplitude in-and-out motion toward the apex. Abrupt pecking motions were avoided. The file was then withdrawn, its flutes were cleaned, and the canal was irrigated. The procedure was then repeated until reaching the working length.

Group II: rotary instrumentation with TruNatomy files (TRN). All files were operated using the X-Smart Plus endodontic motor (Dentsply Sirona, Tulsa, OK), adjusted at 500 rpm/1.5 Ncm following the manufacturer’s instructions.

A TRN Orifice Modifier (20/0.08) was used in the coronal third only, followed by a TRN glider (17/0.02) and the prime instrument (26/0.04). All the instruments were used with two to three gentle 2–5 mm movements into the root canal, the file was then withdrawn, its flutes were cleaned, and the canal was irrigated. The procedure was then repeated until reaching the working length. Both the glider and the prime instrument were used to the full working length.

Group III: instrumentation with WaveOne Gold instruments (WOG). WOG glider file (15/0.02 variable taper) was used. The canals were prepared with the WOG primary instrument (25/0.07) using the X-Smart Plus endodontic motor adjusted for reciprocating motion (170° counter-clockwise and 50° clockwise). The file was used with a slow, in-and-out pecking motion according to the manufacturer’s instructions. This protocol was repeated until the working length was reached. The file was withdrawn after three pecks, its flutes were cleaned, and the canal was irrigated [13].

After each file, the canals were irrigated with 3 ml of distilled water (warmed to 40 °C) with a flow rate of approximately 3 ml/min. All root canals received a final flush of 1 ml of distilled water using a plastic syringe, and a 30-gauge needle tip (NaviTip, Ultradent, South Jordan, UT, USA) positioned 2 mm short of the working length.

Each test instrument was only used to prepare one canal to avoid the carry-over of debris in the file flutes and the process of cleaning the flutes after each use, which could affect the results. The patency of the canals during the instrumentation procedure was maintained with a #10 K file. Instrumentation was considered complete when the file had reached the working length and rotated freely. All root canals in the three groups were instrumented by one operator, whereas the extruded debris was assessed by another examiner blinded to the tested groups.

After instrumentation and irrigation, the separated stoppers with the mesial root were removed from the pre-weighed Eppendorf tubes. The external surfaces of the roots were flushed with 1 ml distilled water to collect debris adhering to external root surfaces. All tubes were then stored at 70 °C in an incubator for five days to evaporate the water before weighing the extruded debris. The apically extruded debris collected in the pre-weighed Eppendorf tubes was weighed again (W2) using the same analytical balance to get the final weight of the tubes containing the extruded debris. Each tube was weighed three times, and the mean value was calculated. The amount of apically extruded debris was then calculated by subtracting the initial weight of the tube from the final weight (W2–W1) [17].

### Statistical analysis

The numerical data were represented as mean and standard deviation (SD) values. The Shapiro–Wilk test was used to test for normality. The homogeneity of variances was tested using Levene’s test. A one-way ANOVA test, followed by Tukey’s post hoc test, was used for intergroup comparisons. The significance level was set at *p* < 0.05 within all tests. Statistical analysis was performed with IBM (IBM Corporation, NY, USA) SPSS (SPSS, IBM Company) Statistics Version 26 for Windows.

## Results

The mean and SD values were calculated and tabulated in Table 1. By comparing the values of the percentage change of weight increase after preparation, it was found that WOG (0.111 ± 0.039) had a statistically significant lower value than TRS (0.274 ± 0.030) and TRN instruments (0.288 ± 0.069) (*p* < 0.001).

**Table 1:** Mean and standard deviation (SD) values for the weight of extruded debris (mg).

Time	Weight of extruded debris (mean ± SD)			*f* value	*p* value
	TRUShape	TruNatomy	WaveOne Gold		
Difference in weight (W2 – W1)	0.274 ± 0.030^A^	0.288 ± 0.069^A^	0.111 ± 0.039^B^	19.945	<0.001*

Different upper superscript letters indicate a statistically significant difference within the same horizontal row.

*Significant (*p* < 0.05).

## Discussion

Literature has reported that the apical extrusion of debris that occurs during chemo-mechanical preparation is the leading cause of inter-treatment flare-ups and postoperative pain after root canal treatment [18]. There are multiple factors that have an impact on the amount of extruded debris, such as design, number, and size of the instruments used in each system, preparation technique, and kinematics [19]. Thus, the aim of the present study was to compare and evaluate TRUShape 3D Conforming files and TruNatomy files versus WaveOne Gold files regarding the amount of apically extruded debris after preparation of mesial root canals of permanent mandibular molars.

Tooth type and curvature influence the amount of extruded debris [20]. Mandibular molars with curved mesiobuccal root canals were used to simulate clinical situations and present the challenges the clinician faces during instrumentation [21]. In addition, the incidences of flare-ups were significantly higher in endodontically treated molars [22]. Efforts were made to balance the samples to decrease the influence of canal anatomy.

Numerous methodologies have been used to assess the amount of debris extruded apically, such as the scoring system and weighing the material using a microbalance. The generally accepted Myers and Montgomery method [16] affords more precise measurements [23]. However, the amount of extruded material is tremendously low, usually in fractions of a milligram. Additional sources that may affect the weight must be considered, such as the influence of touching the assembly by moist fingers or even contamination by contents from the environment where the specimens are kept [6]. In the present study, the apparatus was secured to prevent any direct contacts that could affect the results (Fig. 1). However, it lacks the stimulation of periapical tissue resistance. A simulation of back-pressure of the periapical tissues has been suggested by utilizing floral foam [24], but this setup endures numerous drawbacks such as absorption of the irrigant or debris. Therefore, the present study has made no attempt to simulate the periapical resistance.

Distilled water was utilized in this study for irrigating in all the experimental groups because it lacks the solvent effect of NaOCl. Thus, the extrusion of debris depends only on the mechanical activity of the instruments. In addition, the use of NaOCl leads to the sodium crystallization phenomenon, which could have affected the results of this study [6]. During irrigation, the needle penetrated 2 mm shorter than the working length (passive injection) to avoid the production of high apical pressure, which could lead to increasing the risk for apical extrusion of debris [25].

Environmental temperature impacts NiTi metallurgy and its physical properties [26]. The intra-canal temperature in in vivo conditions was reported to be approximately 35 °C [27]. Thus, all solutions used in the study were warmed to 40 °C prior to the application to sustain the temperature during instrumentation in order to recreate a clinical situation. Since the method of debris collection and its weighing is very critical, the debris collected in vials was stored in an incubator at 70 °C for 5 days until the distilled solution was completely dried to ensure complete moisture elimination and to emphasize that the collected debris was from the mechanical preparation of the root canals.

Results showed that the TRS and TRN files produced significantly more debris than the WOG files (*p* < 0.001). Thus, the null hypothesis was rejected. These results have been confirmed previously as the decreased debris extrusion of reciprocating systems was credited to the balanced force and pressure-less mechanics [19, 28]. Predin Djuric et al. [29] when comparing apical debris extrusion produced by a single-file system used in counter-clockwise reciprocation, clockwise rotation, and clockwise reciprocation, the lowest mean values were recorded by clockwise reciprocation groups.

In fact, contradicting results could be found regarding the instrument kinematics, reciprocation motion was linked with increased debris extrusion [30]. Some studies, including this study, showed that reciprocating systems were associated with less debris extrusion [28, 31]. Reciprocation could be considered as a form of automated force balance technique, allowing better control of debris extrusion toward apical tissues [31]. On the other hand, da Silva et al. [32] showed a comparable amount of apically extruded debris when comparing rotary (ProTaper Universal and TRS) and reciprocating (Reciproc Blue) instruments with no significant differences (*p* > 0.05). The conflicting results of these studies regarding the debris extrusion of reciprocating and continuous rotation rotary instruments may be due to the heterogeneity of research methodologies and materials used [15].

A recent systematic review indicated that the amount of extruded debris is significantly affected by the cross-sectional design of the rotary instrument rather than the motion kinematics [23]. The WOG system has an offset parallelogram-shaped cross-section with two 85° cutting edges contacting the canal wall and a 24° helical angle at the active part of the file, leaving one cutting edge in contact with the canal wall, thus limiting the engagement zone [33]. The extra space around the instrument also gives space for debris removal, which could explain this study’s results. These results are in agreement with those of Çapar and Arslan [34], who stated that files with a rectangular cross-section produced less debris extrusion than those with a triangular cross-section.

Though WOG has a 7% taper in the last file compared to TRS and TRN instruments (6% and 4% taper, respectively), WOG showed significantly lower debris extrusion values than both files. Previous studies showed similar results supporting this study; increasing the instrument taper did not lead to more apical extrusion [35, 36].

Previous studies reported that increasing the number of instruments used may create an additional factor that causes the increased level of debris extrusion [37]. This could be applied in the current study, where the TRN system comprised three files, an orifice modifier, a glider, and the prime instrument (26/0.04), producing significantly more debris than WOG and TRS systems, where both systems are composed of only two files. Current studies evaluating the debris extrusion of TRN instruments showed significantly less debris extrusion than ProTaper Next instruments (Dentsply Maillefer, Ballaigues, Switzerland) [15, 38]. However, a direct comparison with the results of this study was not made due to the different instruments used.

One of the limitations of this study was the use of different rotary systems with different numbers of files, tapers, rotational speeds, and kinematics. However, the aim of this study was to test the effect of minimally invasive canal-shaping systems as a whole on the quantity of apically extruded debris. In-vitro studies could act as a baseline for upcoming clinical studies, not to mention that it provides better, more precise conditions in order to develop consistent comparisons between the tested groups.

## Conclusions

Under the conditions of this study, it can be concluded that debris extrusion is an inevitable consequence of root canal instrumentation. Moreover, in regards to debris extrusion, WaveOne Gold results outperformed TRUShape and TruNatomy instruments in severely curved canals. Future in vivo studies comparing the incidence and intensity of postoperative pain after mechanical preparation is required for further correlation.
